# Pyroptosis-based risk score predicts prognosis and drug sensitivity in lung adenocarcinoma

**DOI:** 10.1515/med-2023-0663

**Published:** 2023-03-13

**Authors:** Zhengsong Jiang, Xiang Wang, Jinghan Huang, Guoyin Li, Shangfu Li

**Affiliations:** Department of Laboratory Medicine, The First Hospital of Jiujiang, Jiujiang, Jiangxi, China; Xiangya School of Medicine, Central South University, Changsha, Hunan, China; St. Croix Lutheran Academy, Minnesota, USA; Key Laboratory of Modern Teaching Technology, Ministry of Education, Shaanxi Normal University, Xi’an, 710061, China; College of Life Science and Agronomy, Zhoukou Normal University, Zhoukou, China; Department of Oncology, Yueyang Second People’s Hospital, Yueyang, Hunan, 414022, China

**Keywords:** pyroptosis, lung adenocarcinoma, tumor microenvironment, tumor mutation burden, drug sensitivity

## Abstract

Pyroptosis is a recently identified form of programmed cell death; however, its role in lung adenocarcinoma (LUAD) remains unclear. Therefore, we set out to explore the prognostic potential of pyroptosis-related genes in LUAD. The pyroptosis-related risk score (PRRS) was developed by least absolute shrinkage and selection operator Cox regression and multivariate Cox regression. We found that PRRS was an independent prognostic factor for LUAD. LUAD patients in the high-PRRS group showed a significantly shorter overall survival (OS) and enriched in cell proliferation-related pathways. Then pathway enrichment analyses, mutation profile, tumor microenvironment, and drug sensitivity analysis were further studied in PRRS stratified LUAD patients. Tumor purity (TP) analyses revealed that L-PRRS LUAD patients had a lower TP, and patients in L-TP + L-PRRS subgroup had the most prolonged OS. Mutation analyses suggested that the L-PRRS LUAD patients had a lower tumor mutation burden (TMB), and patients in H-TMB + L-PRRS subgroup had the most prolonged OS. Drug sensitivity analyses showed that PRRS was significantly negatively correlated with the sensitivity of cisplatin, besarotene, etc., while it was significantly positively correlated with the sensitivity of kin001-135. Eventually, a nomogram was constructed based on PRRS and clinical characters of LUAD. Overall, the pyroptosis-related signature is helpful for prognostic prediction and in guiding treatment for LUAD patients.

## 1 Introduction

Lung cancer (LC) is the leading cause of death among all cancer types, with an estimated 1.8 million deaths annually, which means one in five cancer-related deaths results from it. It is the most common cancer in males and is primarily distributed in Eastern Europe, Eastern Asia, and Southern Europe [1]. LC can be classified into two types: small cell lung carcinoma (SCLC) and non-small-cell lung carcinoma (NSCLC), with a percentage of 15 and 85%, respectively [2]. NSCLC can be further subtyped as squamous-cell carcinoma, adenocarcinoma, and large-cell carcinoma, among which lung adenocarcinoma (LUAD) is the most common, comprising about 40% of all LC [3]. In addition to surgical resection and radiotherapy, systemic treatments for NSCLC include traditional chemotherapy (cytotoxic agents), targeted therapies (tyrosine-kinase inhibitors or TKIs) and immunotherapy (immune checkpoint inhibitors or ICIs) [4,5]. Although great success has been achieved in the clinical application of TKIs and CTLA-4/PD-1/PD-L1 blockers, resistance remains the major challenge. Studies have shown that only 14–45% of patients exhibited significant pathological response when ICI therapy is applied [2,6]. Therefore, indicators that can predict responses to immunotherapy would greatly benefit the effective treatment of NSCLC. Nowadays, the incoming new concept precision medicine has also called for the need to subtype cancer according to molecular features. However, current biomarkers showing the response to ICIs are PD-L1 expression in tumor tissues and tumor mutational burden (TMB), which have inherent shortcomings [7]. Not all patients with a PD-L1 expression proportion higher than the 50% cutoff showed a response to anti-PD-1/PD-L1 antibodies, and some patients below that cutoff expression responded to the treatment [8–10]. Besides, differential expressions within a single lesion also contribute to the discordance between PD-L1 expression and treatment response [11]. Despite NSCLC patients with high TMB being associated with better response to ICIs [12], TMB cannot well predict the overall survival (OS) after ICIs treatment [7]. Hence, new biomarkers that indicate response to ICI treatment and predict prognosis are urgently needed.

Pyroptosis, defined as gasdermin-mediated pro-inflammatory programmed death [13,14], is emerging as a hot topic and has drawn researchers’ interest worldwide. Different from apoptosis or necrosis, pyroptosis has its unique mechanism and characteristics. In the canonical pathway, it begins with the assembly of inflammasome, which takes place after pattern recognition receptors recognize signals from bacteria, viruses, etc. [15]. Subsequently, gasdermins are cleaved by caspases or granzymes. The N-terminal poreforming domain is separated from the C-terminal repressor domain and functions by forming pores in the cell membrane, leading to the release of inflammation mediators, including IL-1β and IL-18, and cell death [16]. Accumulating studies have recognized the two-sided role of pyroptosis in tumorigenesis and cancer progression. Gasdermin D (GSDMD) was found to be up-regulated in NSCLC and promote tumor growth [17]. Besides, both paclitaxel and cisplatin can induce pyroptosis [18], and cisplatin-sensitive NSCLC cells had higher expression of inflammasome components than cisplatin-resistant cells [19]. Moreover, pyroptosis is closely linked with anti-cancer immunity and immunotherapy response [20,21]. Synergistic effects were observed when giving ICIs and gasdermin treatment simultaneously [22].

Given the above facts, it is reasonable to speculate that pyroptosis-related genes (PRGs) might have prognostic values and indicate drug resistance. Over the years, there is a growing body of literature that investigated the application potential of PRGs and constructed different forms of pyroptosis-related risk score (PRRS) in various types of cancer [23–29]. Thanks to high-throughput sequencing, microarray, and establishment of public datasets, it is possible to make a thorough bioinformatics analysis based on the combination of previous data. Therefore, our study aims to utilize data from TCGA and GEO to screen PRGs that are associated with the prognosis of LUAD patients and construct a risk score based on the expression of those genes. Then we analyze the association of the risk score with prognosis, tumor microenvironment (TME), TMB, and drug sensitivity, attempting to broaden the clinical application of the risk score. Eventually, we established a nomogram based on PRRS and clinical characters that can effectively predict the prognosis of LUAD patients.

## 2 Materials and methods

### 2.1 Data acquisition and processing

The LUAD projects of the TCGA (TCGA_LUAD), GSE31210, GSE41271, GSE42127, GSE68465, and GSE72094 datasets were obtained from public databases and were processed as described in our previous study [30]. Briefly, normalized RNA-seq data (HTSeq-FPKM) of the TCGA-LUAD cohort were used for analyses with no further transformation and normalization. The gene expression data (series matrix file) downloaded from the GEO database were normalized (if required) by the normalizeBetweenArrays function of the “limma” package in R. The mutation data of the TCGA_LUAD cohort were downloaded, processed, and visualized as reported in Song’s study [31]. All datasets used in this work were downloaded from public databases, and an extra ethical approval was not necessary.

### 2.2 Development of PRRS

The COX regression analysis was used to identify the PRGs that were significantly related to the prognosis of patients, and patients were divided into C1 and C2 clusters by the consistent cluster analysis according to their expression levels. Patients from the TCGA_LUAD cohort were divided into two clusters, and the “limma” package in R software was used for differential expression analysis between them (log FC ≥ 1, FDR ≤ 0.05). Univariate Cox regression analysis was performed for these differentially expressed genes to generate genes associated with prognosis (*p* < 0.01). The above generated genes were input into the least absolute contraction and selection operator (LASSO) regression mode, which generated 14 key genes, and their corresponding coefficients were obtained by multi-variate cox analysis. A new score for each patient was calculated by the formula as follows: score = ∑_*i*_ Coefficient(Gene *i*) Expression(Gene *i*). To facilitate comparison across different LUAD cohorts, the PRRS was calculated with the formula as follows: PRRS = (score-Min)/absolute (Max) [32,33]. The TCGA_LUAD cohort was used as the training set, and GSE31210, GSE41271, GSE42127, GSE68465, and GSE72094 cohorts were used as the validation sets.

### 2.3 Enrichment analysis

In the TCGA_LUAD cohort, a total of 128 differentially expressed genes were identified between high-PRRS (H-PRRS) and low-PRRS (L-PRRS) subgroups. Gene ontology (GO) and Kyoto Encyclopedia of Genes and Genomes (KEGG) analysis of the 128 genes in the TCGA_LUAD cohort were performed by the “clusterProfiler,” “org.Hs.eg.db,” “DOSE,” and “enrichplot” packages in R software [34]. Gene Set Enrichment Analysis (GSEA) of PRRS-based classification of LUAD patients was performed by “c2.cp.kegg.symbols.gmt” package in R software [32].

### 2.4 Immune profile and mutation profile analysis

TME score of patients from the TCGA_LUAD cohort was calculated using the “estimate” package in R software. The infiltration ratio of 22 types of immune cells in TME was calculated by the CIBERSORT algorithm in R software [35]. The TMB and the mutant-allele tumor heterogeneity score were calculated by the package “maftools” in R software [36].

### 2.5 Drug sensitivity analysis

Immunotherapy data of patients in the TCGA_LUAD cohort were downloaded from The Cancer Immunome Atlas (https://tcia.at/). Drug sensitivity analysis was performed by the “prrophetic” package in R software.

### 2.6 Development and evaluation of the nomogram

Univariate and multivariate Cox regression analyses were performed using the “survival” package in R. The nomogram was performed using the “rsm” package in R. Calibration curve was used to evaluate the accuracy of the nomogram.

### 2.7 Statistical analysis

The data were analyzed by R software (version 4.1.0). The “limma” package was used for differential expression analysis between the two clusters. The “limma,” “survival,” and “ConsensusClusterPlus” packages were used for the consistent cluster analysis. The univariate Cox regression analysis was performed by the “survival” package. The LASSO regression model was developed by “glmnet” and “survival” packages. Survival analysis was executed by “survival” and “survminer” packages. ROC curves were drawn by “survival,” “survminer,” “timeROC,” and “rms” packages. The C-index value was calculated by “dplyr,” “survival” “rms,” and “pec” packages. The nomogram was drawn by “survival,” “regplot,” “survminer,” “timeROC,” and “rms” packages. A value of *p* < 0.05 was considered to be statistically significant (*, *p* < 0.05; **, *p* < 0.01; ***, *p* < 0.001).

## 3 Results

### 3.1 The construction and predictive analysis of pyroptosis-related LUAD subtypes

**Figure 1:**
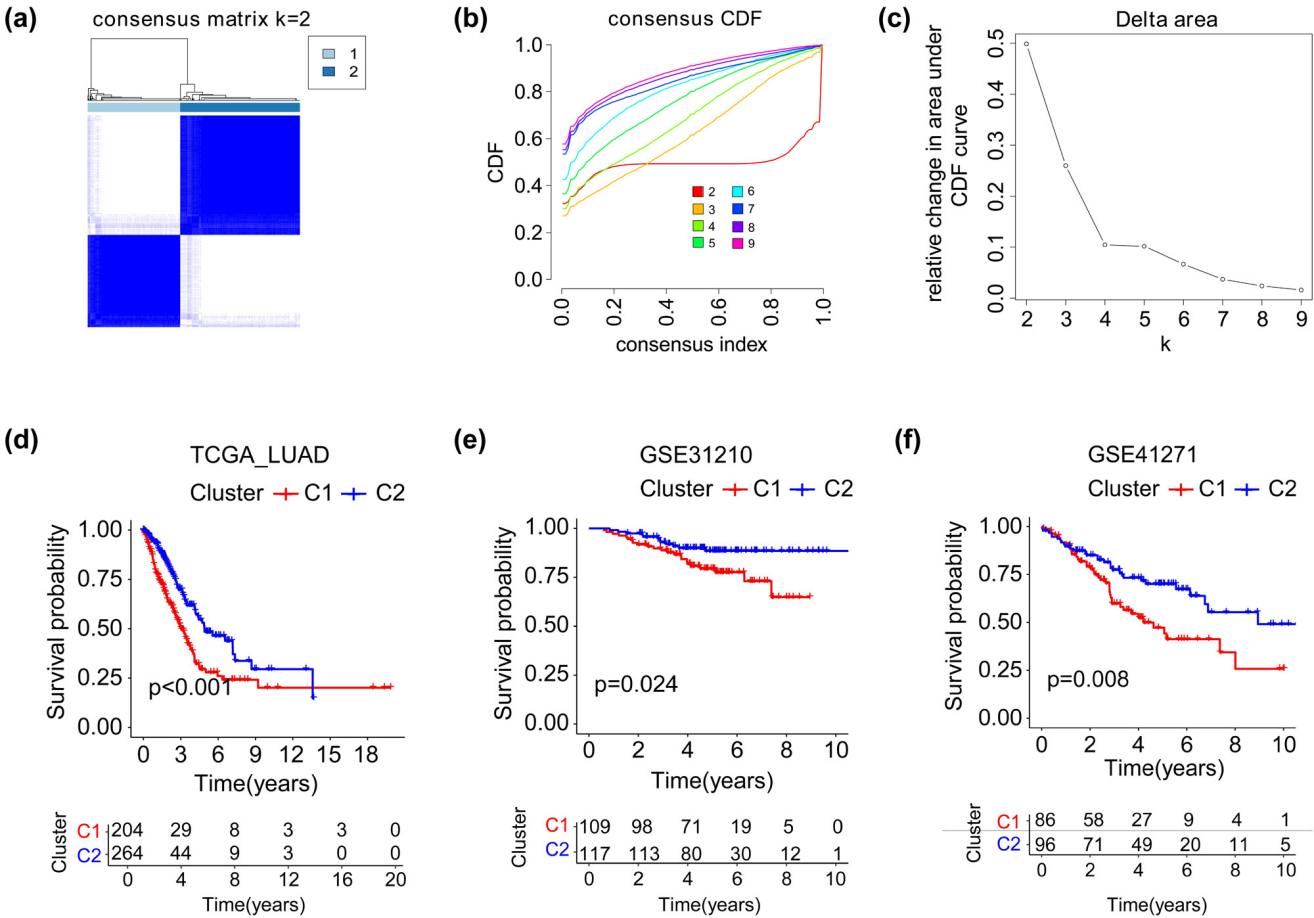
Classification and prognosis of LUAD according to the PRGs. (a) Two clusters were generated by unsupervised consensus clustering. (b and c) Consensus clustering cumulative distribution function (CDF) and relative change in area under the CDF curve (*k* from 2 to 10). (d–f) KM analysis showed that patients in the C2 cluster had a longer OS in the TCGA_LUAD (d), GSE31210 (e), and GSE41271 (f) cohorts.

According to Hu’s study [37], we screened 52 PRGs in the TCGA_LUAD dataset (Figure S1; Table S1) and found that 17 genes were down-regulated and 26 genes were up-regulated in tumor tissues compared to normal tissues (Figure S1; Table S2; *p* < 0.05). This result revealed that the expression of PRGs was dysregulated in LUAD. Subsequently, we performed a consistent cluster analysis using 52 PRGs on patients from the TCGA_LUAD cohort. To confirm that PRG can effectively distinguish patients, we increased the clustering variable (*k*) from 2 to 10. The results showed that at *k* = 2, the intragroup correlations were low, indicating that cases could be well split into two categories (Figure 1a–c). According to the PRGs, patients from TCGA_LUAD, GSE31210, and GSE41271 cohorts were divided into two clusters, respectively. Kaplan–Meier (KM) analysis suggested that patients in the C2 cluster had a more prolonged OS time in all three cohorts (Figure 1d and f). These results suggested that the expression level of PRGs was closely related to the prognosis of LUAD patients.

### 3.2 Construction and validation of PRRS

**Figure 2:**
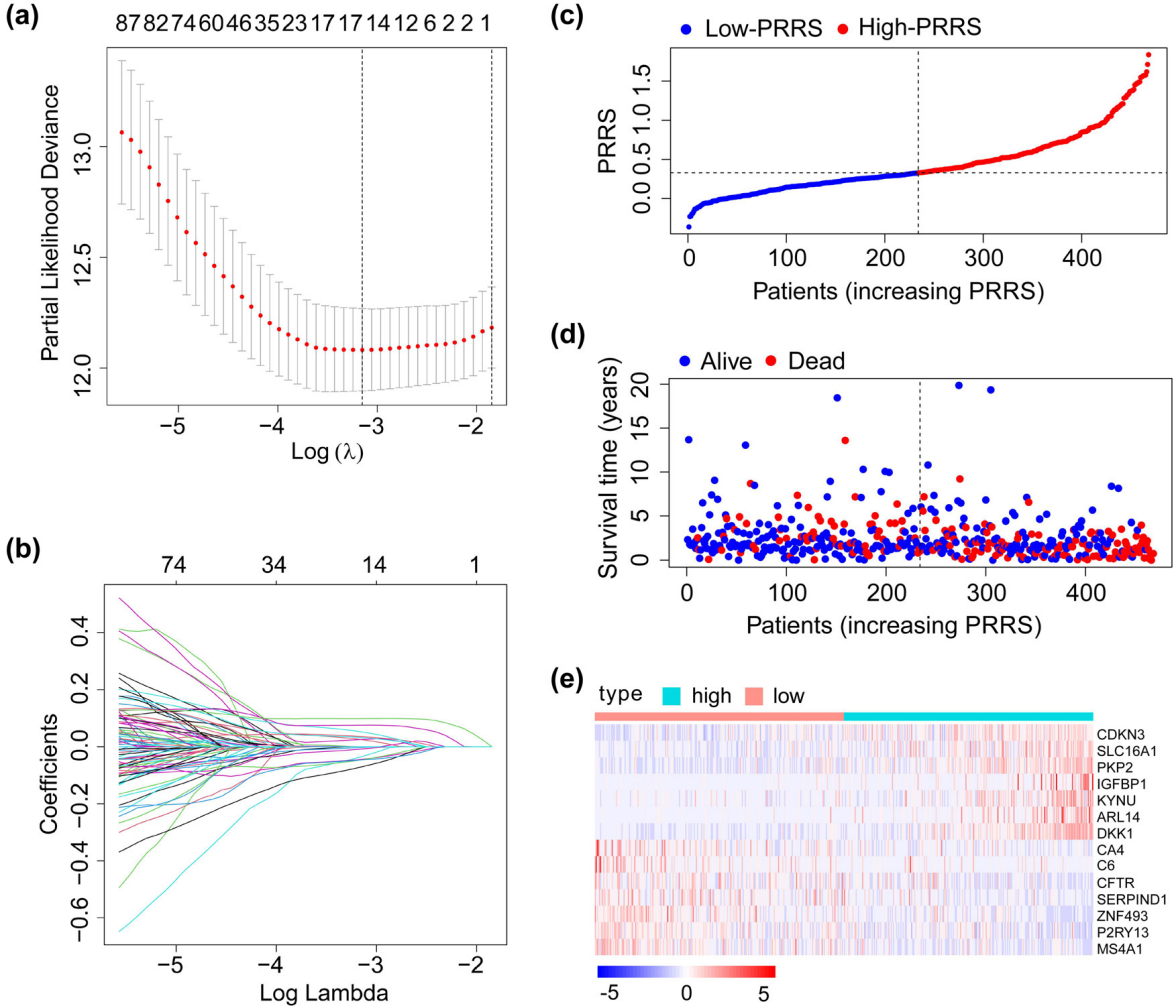
Construction of PRRS using TCGA_LUAD dataset. (a and b) LASSO Cox regression model was constructed from 125 prognosis-related genes. The 14 crucial genes were generated by the best-fit profile. (c) Distribution and cutoff value of the PRRS. (d) OS and survival status of patients in H-PRRS and L-PRRS groups. (e) Expression heatmap of the 14 crucial genes in the TCGA_LUAD dataset.

Patients from the TCGA_LUAD cohort were used as the training set to develop the pyroptosis-related risk models. First, patients were classified into C1 and C2 clusters according to PRGs. Second, we performed differential gene analysis on the two clusters and generated 567 differentially expressed genes (log FC > 1; FDR < 0.05). Third, we carried out a univariate Cox regression analysis on the 567 genes and identified 125 genes with significant prognostic correlation (*p* < 0.01). Finally, the 125 genes were put into a LASSO regression model and obtained 14 crucial genes and their corresponding coefficients (Figure 2a and b). The score of each patient in a cohort was calculated by the following formula: score = 0.0247 * SLC16A1 + 0.0355 * ARL14 − 0.0081 * CFTR + 0.0251 * CDKN3 − 0.0026 * SERPIND1 + 0.052 * IGFBP1 − 0.0351 * CA4 − 0.0443 * P2RY13 − 0.0708 * C6 − 0.0462 * ZNF493 + 0.0753 * PKP2 + 0.0982 * DKK1 − 0.0466 * MS4A1 + 0.0319 * KYNU. The PRRS of patients was calculated as reported in Section 2.

**Figure 3:**
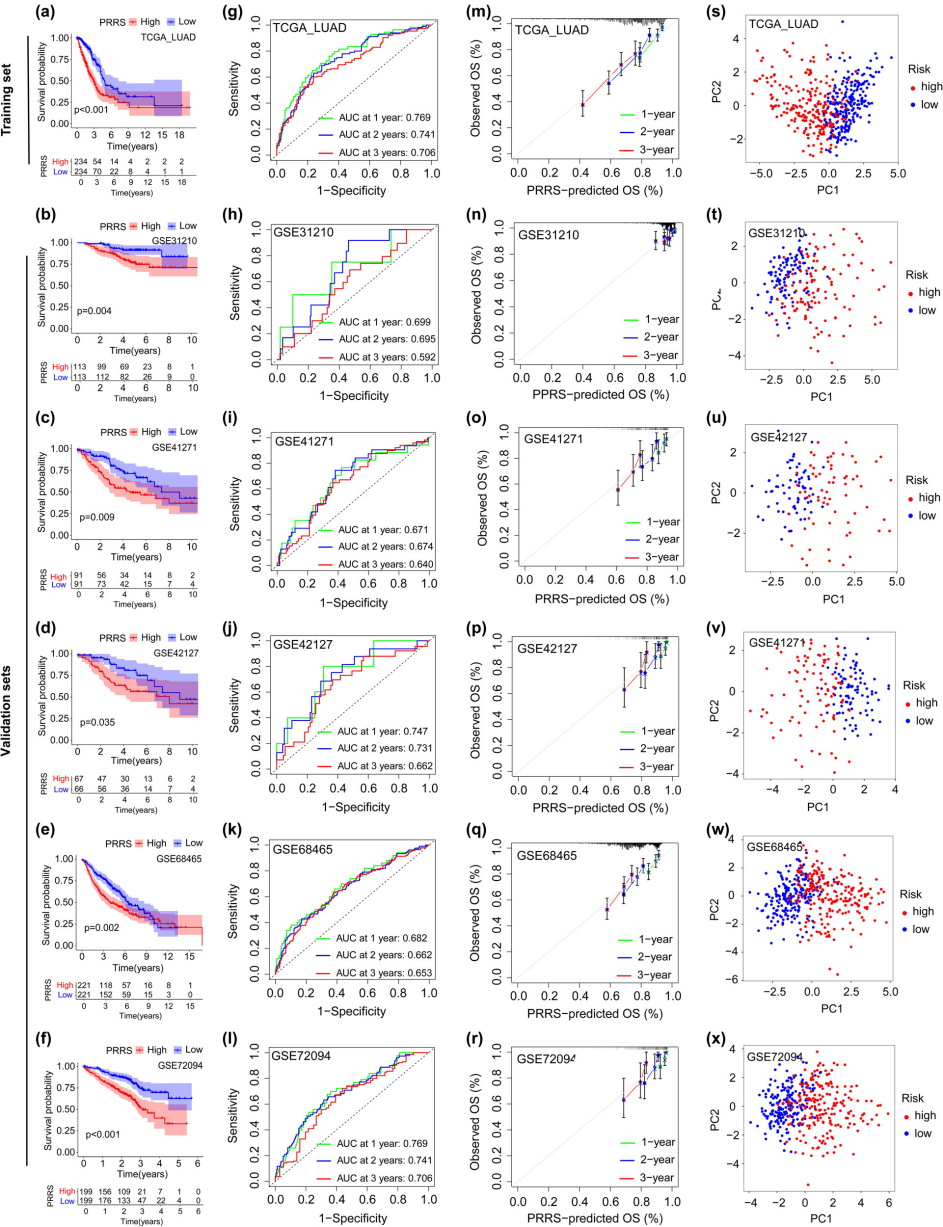
Evaluation of the effectiveness of PRRS in the training and verification datasets. (a–f) KM survival curves of OS in training (a) and validation (b–f) datasets. (g–l) ROC curves evaluate the effectiveness of PRRS in training (g) and validation (h–l) datasets. (m–r) Calibration curves for evaluating the accuracy of PRRS in training (m) and validation (n–r) datasets. (s–x) PCA results of patients from the training (s) and validation (t–x) datasets according to PRRS.

Patients were equally assigned to H-PRRS and L-PRRS groups (Figure 2c and d). KM analysis showed that patients from the L-PRRS had significantly longer OS (Figure 3a). The AUC values of PRRS in the TCGA_LUAD dataset were 0.769 for 1 year, 0.741 for 2 years, and 0.706 for 3 years (Figure 3g). In the five external validation datasets (GSE31210, GSE41271, GSE42127, GSE68465, and GSE72094 cohorts), patients were equally divided into H-PRRS and L-PRRS groups based on the value of PRRS in each cohort. In all of the five validation cohorts, patients from the L-PRRS groups had significantly longer OS than that in the H-PRRS groups, which was highly consistent with the training cohort (Figure 3b–f). The area under curve (AUC) values of PRRS in the GSE31210 cohort were 0.699 for 1 year, 0.695 for 2 years, and 0.592 for 3 years; were 0.671 for 1 year, 0.674 for 2 years, and 0.64 for 3 years in the GSE41271 cohort; were 0.747 for 1 year, 0.731 for 2 years, and 0.662 for 3 years in the GSE42127 cohort; were 0.682 for 1 year, 0.662 for 2 years, and 0.653 for 3 years in the GSE68465 cohort, and were 0.769 for 1 year, 0.741 for 2 years, and 0.706 for 3 years in the GSE72094 cohort (Figure 3h–l). The calibration curves confirmed that the PRRS could reasonably predict the prognosis of patients in both the training and validation cohorts (Figure 3m–r). The principal component analysis (PCA) results showed that PRRS could effectively distinguish H-PRRS and lL-PRRS patients in both the training and validation datasets (Figure 3s–x). The aforementioned results confirmed that PRRS was an excellent prognostic indicator of LUAD.

**Figure 4:**
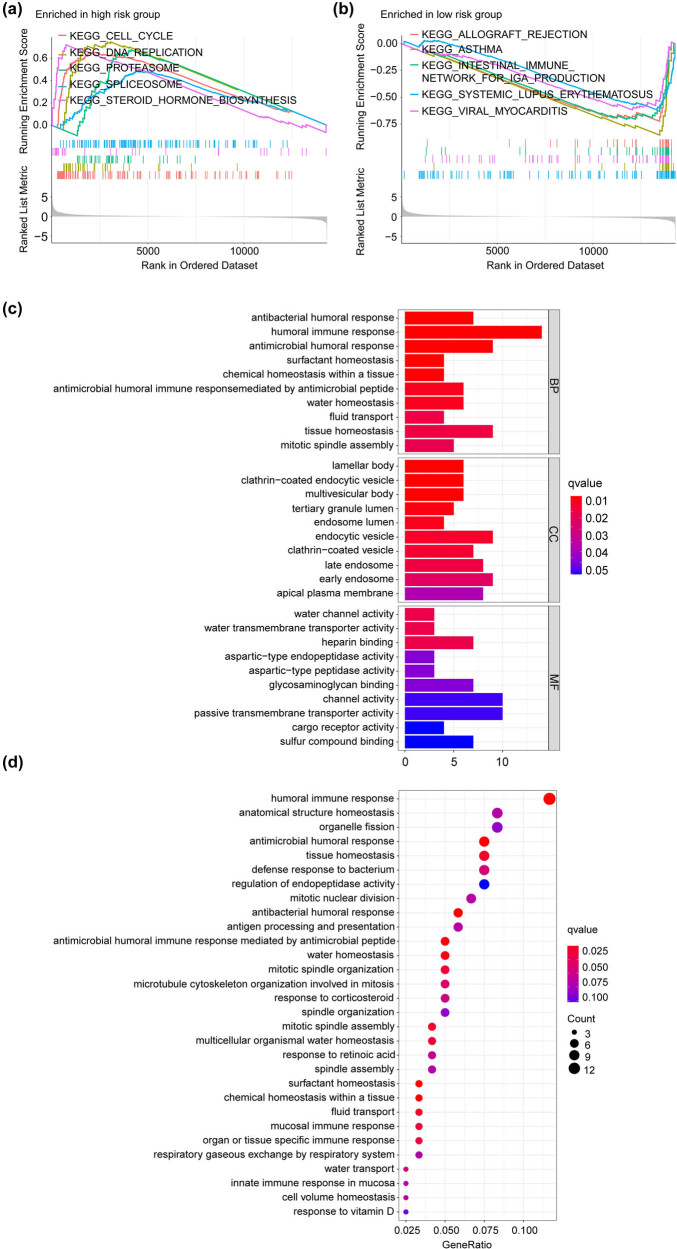
Enrichment analysis of PRRS-based LUAD groups. (a and b) GSEA of the H-PRRS and L-PRRS groups in the TCGA_LUAD cohort. (c and d) GO and KEGG enrichment analysis of 128 differentially expressed genes between the H-PRRS and L-PRRS groups in the TCGA_LUAD cohort.

To investigate the potential molecular mechanism of prognosis difference between H-PRRS and L-PRRS subgroups, we performed GSEA using the TCGA_LUAD dataset. The results suggested that the H-PRRS subgroup was enriched in cell cycle, DNA replication, proteasome, spliceosome, and steroid hormone biosynthesis pathways. In contrast the L-PRRS subgroup was enriched in allograft rejection, asthma, intestinal immune network for IgA production, systemic lupus erythematosus, and viral myocarditis pathways (Figure 4a and b). We obtained 128 differentially expressed genes (log FC > 1; FDR < 0.05) from H-PRRS and L-PRRS groups using the “limma” package, and performed GO and KEGG analysis. GO and KEGG analysis results suggested that the above differentially expressed genes were mainly enriched in humoral immune response-related pathways, and regulated humoral response (Figure 4c and d).

### 3.3 TME landscape of PRRS-based classification

**Figure 5:**
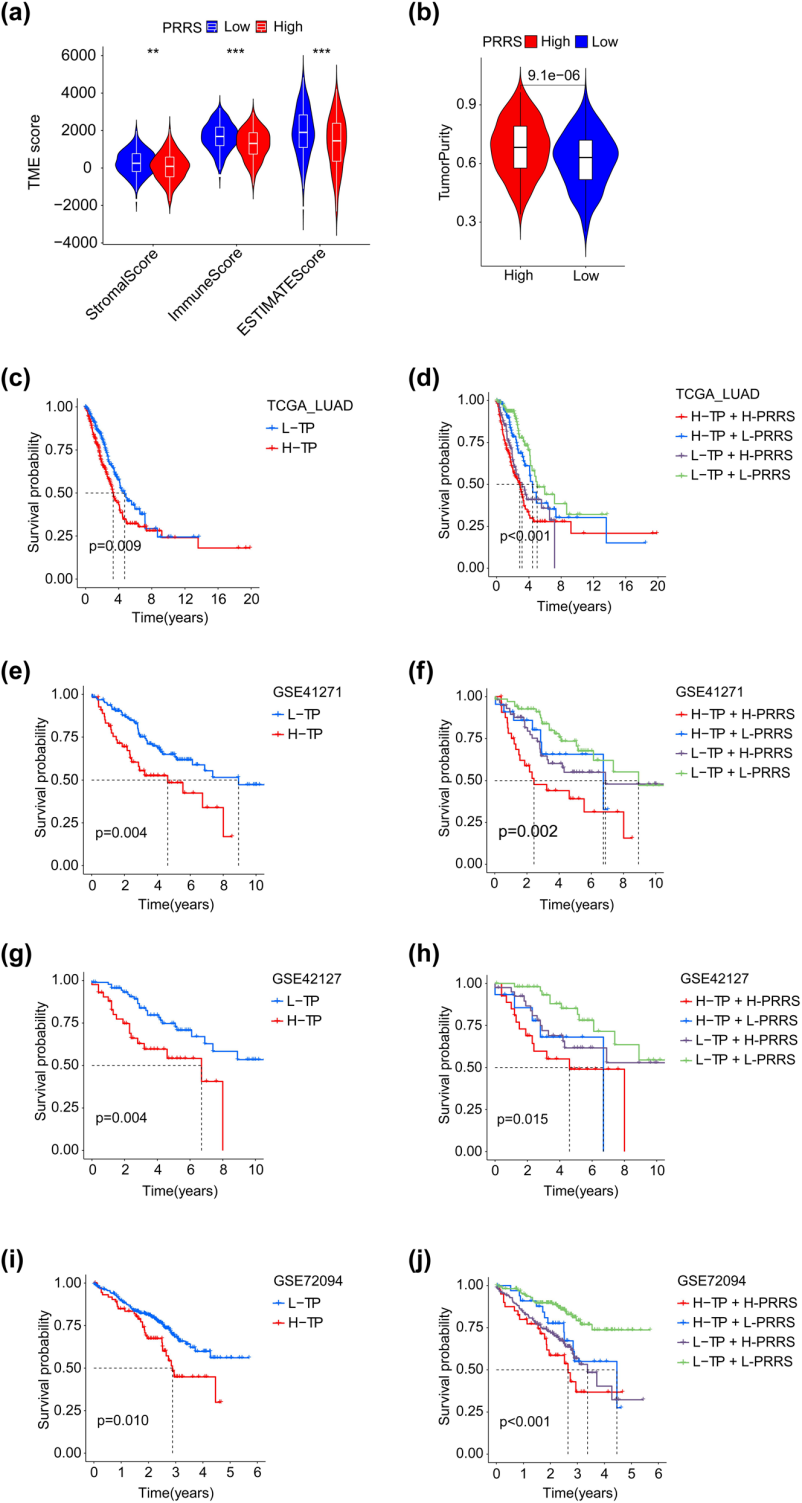
Tumor purity combined with PRRS to evaluate the prognosis of LUAD patients. (a) The immune, stromal, and ESTIMATE scores of the L-PRRS group were higher than the H-PRRS group (*p* < 0.01). (b) The tumor purity of the L-PRRS group was lower than the H-PRRS group (*p* < 0.001). (c, e, g, i) In the TCGA_LUAD (c), GSE41271 (e), GSE42127 (g), and GSE72094 (i) cohorts, patients in the L-TP groups had a longer OS than the H-TP group (*p* ≤ 0.01). (d, f, h, j) In the TCGA_LUAD (d), GSE41271 (f), GSE42127 (h), and GSE72094 (j) cohorts, patients in the L-TP + L-PRRS groups had the best prognosis, while the H-TP + H-PRRS had the worst prognosis. **, *p* < 0.01; ***, *p* < 0.001; L-TP: low tumor purity; H-TP: high tumor purity; L-PRRS: low PRRS; H-PRRS: high PRRS.

TME provides a favorable environment for tumor progression and is closely related to the treatment and prognosis of various tumors [38]. Tumor purity can act as a powerful prognostic indicator for various carcinomas [39–41]. In the TCGA_LUAD cohort, we found that the stromal score or the immune score of patients in the H-PRRS subgroup was significantly lower than that in the L-PRRS subgroup, while the tumor purity was the opposite (Figure 5a and b). We noticed that in the TCGA_LUAD, GSE41271, GSE42127, and GSE72094 cohorts, the infiltration ratios of immune cells such as aDCs, B cells, iDCs, mast cells, neutrophils, T helper cells, and TIL significantly reduced in the H-PRRS subgroup (Figure S1a–d). In addition, correlation analysis confirmed that T cells CD4 memory resting/activated, macrophages M0/M1, dendritic cells resting/activated, and B cells memory were significantly co-expressed with most of the 14 crucial genes (Figure S2).

In the above four datasets, we also identified that the function of HLA and type II IFN response was significantly down-regulated in the HPRRS subgroup (Figure S3a–d). In the four datasets, KM analysis showed that the OS of patients in the low tumor purity (L-TP) group was significantly longer than that in the high tumor purity (H-TP) group (Figure 5c, e, g, and i). In the four cohorts, we comprehensively analyzed PRRS and tumor purity and found that patients in L-TP + L-PRRS group had the best prognosis, and patients in H-TP + H-PRRS group had the worst prognosis (Figure 5d, f, h, and j). The above results suggested that PRRS was closely related to tumor purity and patient prognosis.

### 3.4 Tumor mutation burden (TMB) of PRRS-based classification

**Figure 6:**
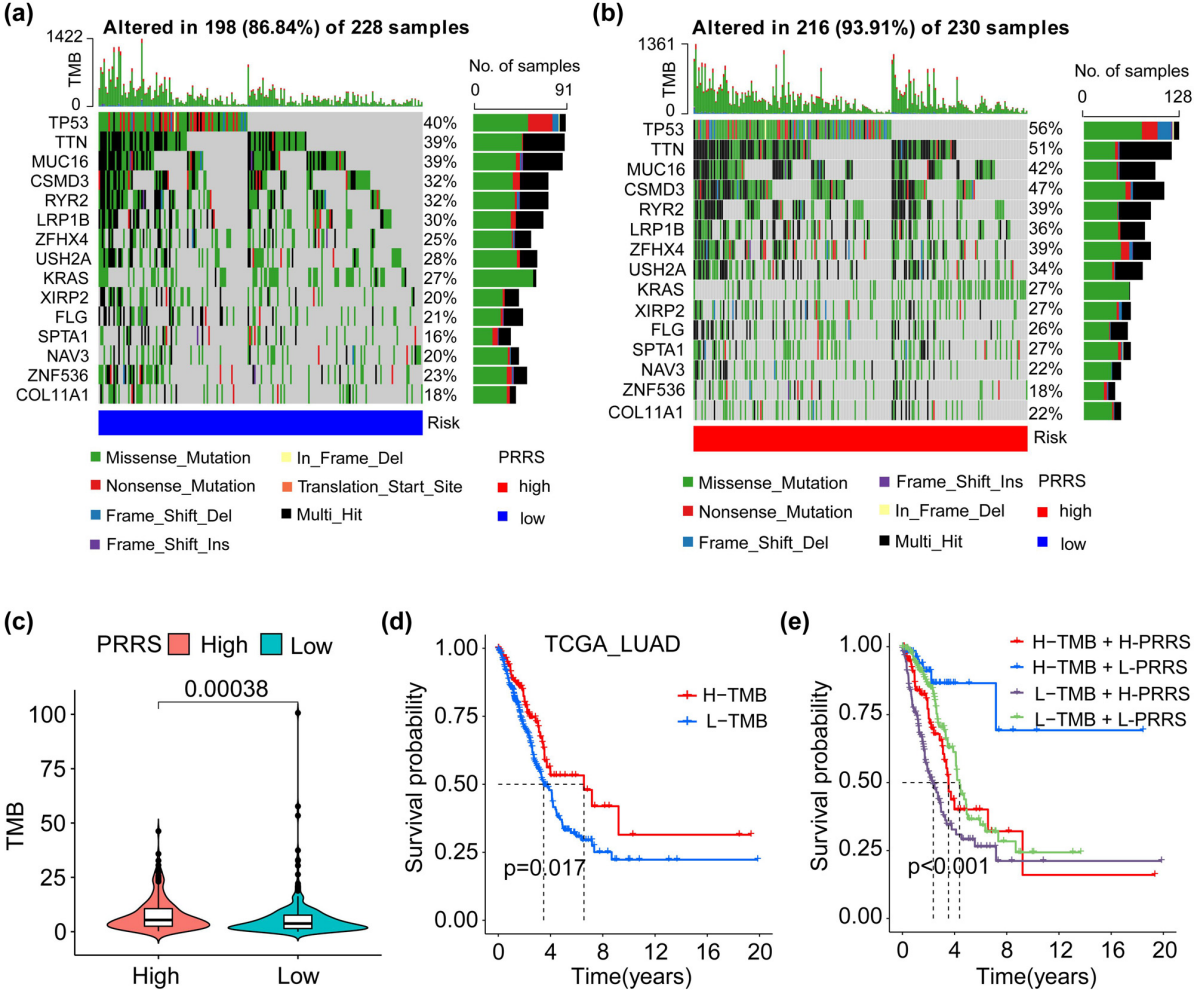
Mutation signatures of PRRS-based LUAD patients. (a and b) Waterfall plots of mutation genes in L-PRRS (a) and H-PRRS (b) subgroups from the TCGA_LUAD cohort. (c) TMB of the H-PRRS group was significantly higher than the L-PRRS group. (d) In the TCGA_LUAD cohorts, patients in the H-TMB groups had a longer OS than the L-TMB group. (e) In the TCGA_LUAD cohorts, patients in the H-TMB + L-PRRS group had the best prognosis. L-TMB: low TMB; H-TMB: high TMB; L-PRRS: low PRRS; H-PRRS: high PRRS.

Previous studies have shown that TMB can be used as a predictor of immunotherapy response in NSCLC [42–44]. We further studied the mutation profile of PRRS-stratified LUAD patients. In the TCGA_LUAD cohort, we observed that the L-PRRS group had a lower TMB, and patients exhibited different mutation signatures between the two subgroups (Figure 6a–c). The top five genes with the highest mutant frequency in the L-PRRS group were TP53 (40%), TTN (39%), MUC16 (39%), CSMD3 (32%), and RYR2 (32%); whereas, those in the H-PRRS group were TP53 (56%), TTN (51%), MUC16 (42%), CSMD3 (47%), and RYR2 (39%) (Figure 6a and b). KM analysis showed that the OS of patients in H**-**TMB group was longer than that of patients in the L**-**TMB group, and patients in the H**-**TMB + L**-**PRRS group had the best prognosis (Figure 6d and e).

### 3.5 Guidance of PRRS in LUAD therapy

**Figure 7:**
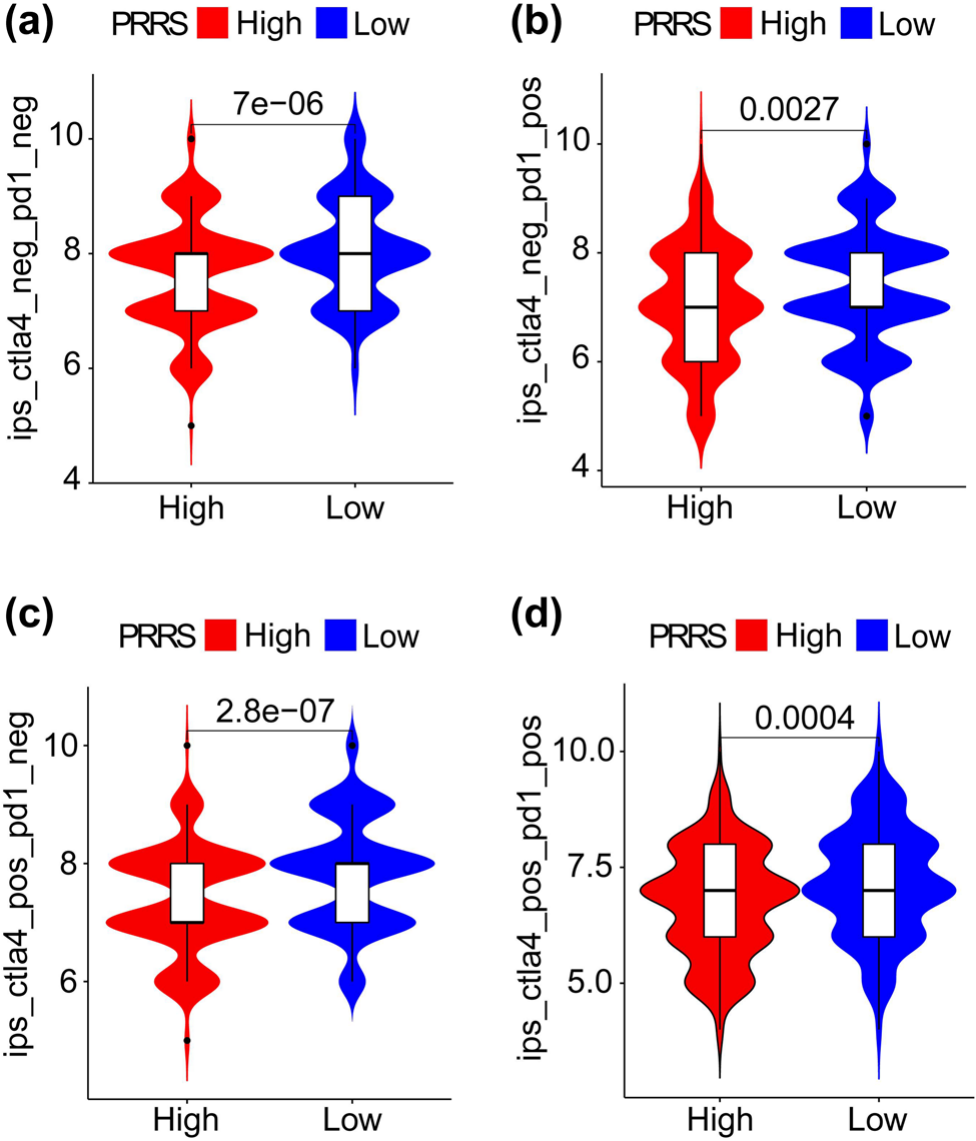
Guidance of PRRS in LUAD immunotherapy. (a–d) LUAD patients in the L-PRRS will benefit more from CTLA4 and, or PD1 inhibitor treatment.

**Figure 8:**
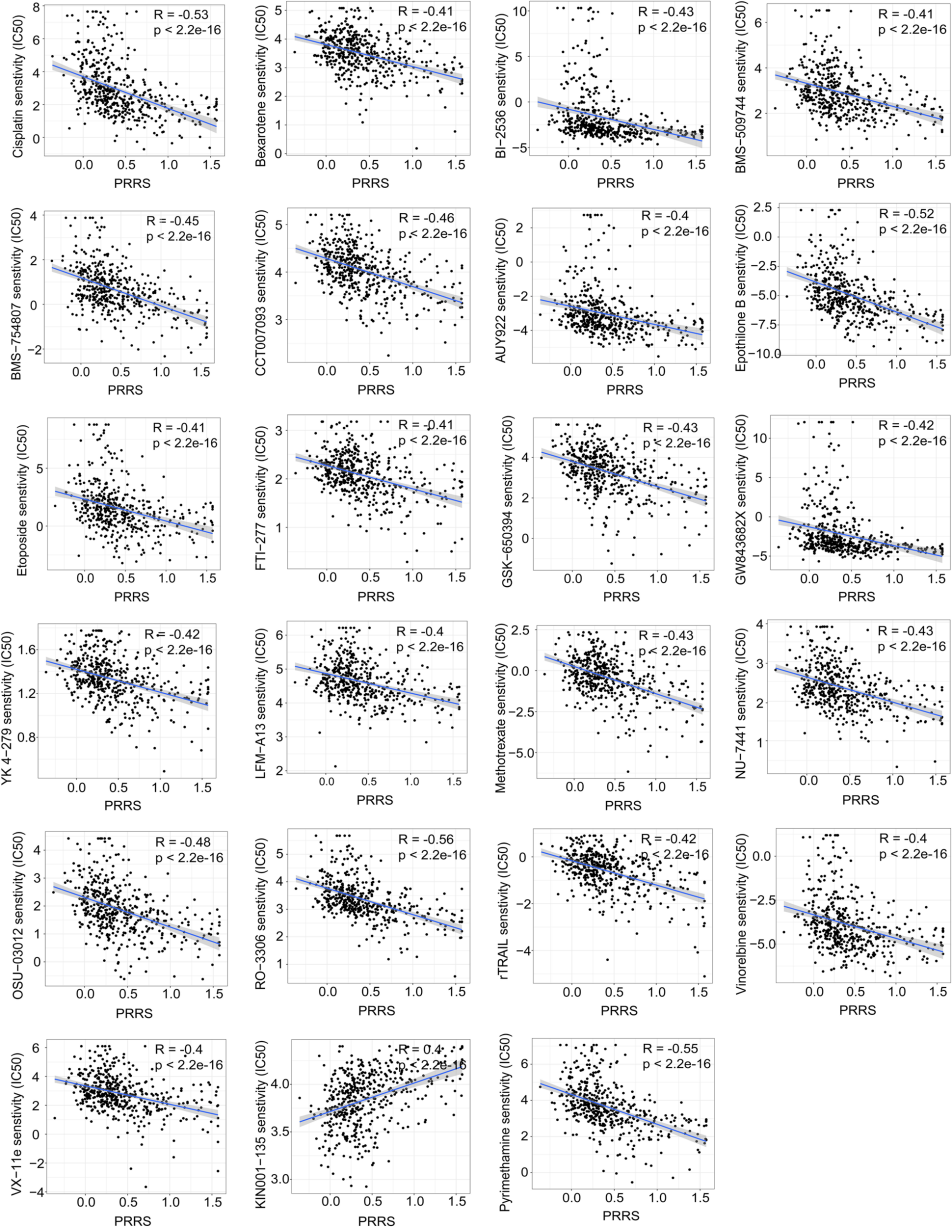
Screening of potential drugs for LUAD patients. Correlation analysis between the sensitivity of 23 drugs and PRRS.

As negative regulators of T cell immunity, CTLA**-**4 and PD-1 have become immunotherapeutic targets for NSCLC. CTLA**-**4 and PD**-**1 negatively regulate T cell activity at different stages of immune response, respectively [45]. We obtained the clinical data of LUAD patients treated with CTLA**-**4 or/and PD**-**1 from The Cancer Immunome Atlas (TCIA) database. We found patients in the L**-**PRRS subgroup could benefit more from immunotherapy (Figure 7a–d). In addition, we also analyzed the correlation between the sensitivity of 23 drugs and PRRS. As shown in Figure 8, the sensitivity of 22 drugs was negatively correlated with PPRS, such as cisplatin, bexarotene, and methotrexate, while the sensitivity of KIN001-135 was positively correlated with PRRS (|*R*| ≥ 0.4; *p* < 0.001). The statistical results suggested that patients in the L-PRRS subgroup had higher sensitivity to 22 drugs, such as cisplatin, bexarotene, and methotrexate, and lower sensitivity to KIN001-135 (*p* < 0.001; Figure S4).

### 3.6 Establishment of a nomogram based on PRRS and clinical characters

**Figure 9:**
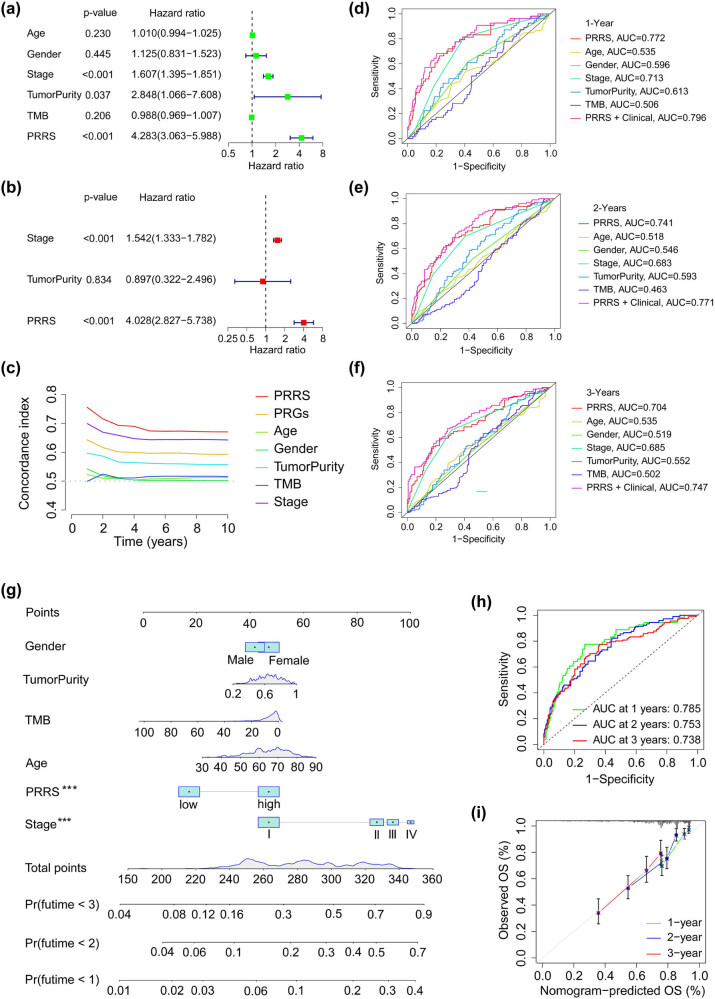
Development and verification of nomogram. (a and b) Univariate and multivariate regression analyses of the correlation between PRRS and clinical characteristics regarding OS in the TCGA-LUAD cohort. (c) C-index curves of PRRS and clinical features. (d–f) Time-dependent ROC analyses of PRRS and, or clinical features regarding the OS and survival status in the TCGA_LUAD cohort. (g) Nomogram is based on gender, age, tumor purity, TMB, PRRS, and stage. (h) Time-dependent ROC analyses of the nomogram regarding the OS and survival status in the TCGA_LUAD cohort. (i) Calibration curves of the nomogram between predicted and observed 3-, 5- and 10-year OS in the TCGA_LUAD cohort.

Univariate and multivariate Cox regression analyses were presented on the TCGA_LUAD dataset, and PRRS, stage, and tumor purity were recognized as independent risk factors for LUAD (Figure 9a and b). We plotted the C-index curves of the above characters, and the found PRRS had the most immense value, indicating that it had the highest prognostic accuracy for LUAD prognosis (Figure 9c). We also drew 1-, 2-, and 3-year ROC curves using PRRS and clinical features, and found that the PRRS + clinical group always had the biggest AUC (Figure 9d and f). Finally, we plotted the nomogram using the above features for LUAD to develop a nomogram to quantitatively establish the 1-, 2- and 3-year survival rates (Figure 9g). TheAUC values of PRRS in the TCGA_LUAD cohort were 0.785 for 1 year, 0.753 for 2 years, and 0.738 for 3 years (Figure 9h). In addition, the calibration curves of patients at 1, 2, and 3 years confirmed the accurateness of the nomogram (Figure 9i). Thus, nomogram was the best model to predict the prognosis of LUAD compared with single risk factor.

## 4 Discussion

Pyroptosis, a novel type of programmed cell death, was first discovered in 1992 [46] but did not catch researchers’ attention until recent years. Pyroptosis is characterized by gasdermin cleavage, pore formation, cell swelling, and subsequent release of inflammatory mediators [20]. There is a growing body of literature that recognizes the two-sided role of pyroptosis in tumorigenesis and cancer progression [47]. On the one hand, pyroptosis is accompanied by IL-1β and IL-18 release, which could mediate tumor-promoting inflammation [48,49]. Gao et al. found that GSDMD was significantly up-regulated in NSCLC and that knockdown of GSDMD mitigated cell proliferation and tumor growth in xenograft mouse models [17]. On the other hand, pyroptosis inhibits tumor progression and stimulates anti-cancer immunity [50]. Previous research studies show that GSDMD is required for CD8+ T cell cytotoxicity toward LC cells [51], and GSDME represses tumor growth *in vivo* [52]. Considering the pivotal role that pyroptosis plays in cancer progression, we suppose that PRGs might have predictive value and be associated with drug resistance in LUAD. Nevertheless, relevant research studies are relatively scarce. Hence, we set out to investigate the predictive potential of PRGs and construct a PRRS for clinical application.

First, we performed cluster analysis using the expression profile of 52 PRGs and split the patients from TCGA_LUAD, GSE31210, and GSE41271 cohorts into two clusters, respectively. In all cohorts, patients in C2 had a better prognosis than those in C1, confirming the predictive potential of PRGs. Then we tried to construct a PRRS more stable than cluster analysis. A total of 567 differentially expressed genes were discovered after comparing the two clusters of the TCGA_LUAD cohort. Further, univariate Cox regression analysis and LASSO regression model extracted 14 crucial genes, and incorporated into the formula to obtain the PRRS.

Among the 14 genes, some have been shown to participate in LC progression or have prognostic potential. ARL14, an ADP ribosylation factor family member, was up-regulated in NSCLC tissue samples, indicating poor survival [53]. ARL14 could promote LUAD cell proliferation, and knockdown of ARL14 induced a dormant state in cancer cells [54]. The cystic fibrosis transmembrane conductance regulator (CFTR) was down-regulated in NSCLC tissues compared with paired normal tissues [55]. High CFTR was correlated with better survival in NSCLC patients, and knockdown of CFTR enhanced cell migration, invasion *in vitro*, and metastasis *in vivo* [56]. High expression of cyclin-dependent kinase inhibitor 3 (CDKN3) was observed in LC cell lines and was associated with poor survival of LUAD patients [57]. Combretastain A4 (CA4) was a tumor suppressor which inhibited NSCLC cell proliferation and tumor growth in xenograft mouse models [58]. Plakophilin 2 (PKP2) was up-regulated in LUAD tissues and LC cells, and its high expression indicated worse prognosis for LUAD patients. Mechanistically, PKP2 promoted LC cell proliferation and invasion via enhancing epithelial–mesenchymal transition (EMT) and focal adhesion [59]. Moreover, PKP2 contributed to LC radioresistance and its high expression was associated with worse survival in LC patients after radiotherapy [60]. The dickkopf WNT signaling pathway inhibitor 1 (DKK1) also exhibited tumor-promoting phenotype in LC. It was up-regulated in NSCLC tissues compared with normal lung tissues and could promote migration, invasion, and EMT in LC cells. Patients with DKK1-positive tumors had shorter disease-free survival than those with negative tumors [61,62]. Notably, the knockdown of DKK1 sensitized NSCLC cell lines to cisplatin treatment, indicating that DKK1 partly contributed to the intrinsic cisplatin resistance [63]. A study revealed that kynureninase (KYNU) expression was positively correlated with CD8+ tumor infiltrating lymphocytes and PD-L1 cell positivity. Higher expression of KYNU was associated with worse OS in LUAD patients [64]. Therefore, our PRRS seems reliable since many of the 14 genes play an active role in LC progression and have prognostic value alone.

Afterward, we investigated the association of PRRS with patient survival, TME, TMB, and drug sensitivity. In both the training and validation cohorts, patients in the L-PRRS groups had significantly longer OS than those in the H-PRRS groups, confirming the predictive value of PRRS in LUAD. Tumor purity reflects the tumor cell content in the tissue and is a prognostic indicator in various cancers [39–41]. Our result revealed that tumor purity of patients in the H-PRRS group was significantly higher than that in the L-PRRS group. Tumor purity can predict prognosis of LUAD patients alone or combined with PRRS. Besides, the infiltration ratios of immune cells such as aDCs, B cells, iDCs, mast cells, neutrophils, T helper cells, and TIL were significantly reduced in the H-PRRS group, suggesting that PRRS is associated with TME in LUAD. TMB is a biomarker for predicting the clinical benefit from immunotherapy response, and higher TMB was associated with prolonged OS after immunotherapy [7,42,44,65]. Our results showed that patients in the H-PRRS group had a higher TMB, suggesting that this group is likely to have survival better after immunotherapy. After analyzing the IPS score acquired from TCIA database, we found that patients in the L-PRRS group had higher IPS than those in the H-PRRS group, suggesting that the L-PRRS group might exhibit a better response to immune checkpoint blockers [66]. Previous research showed that applying gasdermin could sensitize breast cancer cells to anti-PD1 therapy, which supports our results [22].

The underlying mechanism behind the association between pyroptosis and immunotherapy can be rather complex. On the one hand, pyroptosis can secrete IL-1β and IL-18 to trigger inflammatory responses and recruiting immune cells, which might enhance the anti-tumor effects of immunotherapy [67]. In addition, pyroptosis also plays a role in the activation and functioning of immune cells. For instance, the expression of GSDMD is higher in activated CD8+ T cells than in naïve T cells, and GSDMD is essential in the cytolytic ability of CD8+ T cells [68]. On the other hand, pyroptosis is accompanied by the release of inflammatory mediators, such as IL-1 and IL-18, which might facilitate cancer development and progression [48,49]. Moreover, chimeric antigen receptor (CAR) T cell therapy can induce pyroptosis in target cells, and subsequent cytokine release can induce severe adverse reactions after CART therapy [21,69].

Interestingly, PRRS was also associated with the sensitivity of 23 drugs, including cisplatin and other targeted therapies. This is not surprising since chemotherapy drugs like cisplatin can induce pyroptosis in GSDME-high cancer cells and GSDME-deficient mice showed fewer adverse effects induced by chemotherapy [70]. Taken together, PRRS is a powerful predictor of prognosis, immunotherapy response, and drug sensitivity, which might be suitable for clinical application. Eventually, we built a nomogram based on gender, age, tumor purity, TMB, PRRS, and stage, which was accurate for predicting LUAD prognosis.

Pyroptosis-related prognostic signatures have been constructed in various tumors, including glioma, breast cancer, gastric cancer, LC, hepatocellular carcinoma, cervical cancer, etc. [23,25,29,71–78]. However, the PRRS seems more reliable since we included more PRGs (52 genes) as the input than the other studies. Besides, we used a combination of univariate Cox regression analysis and LASSO regression model to obtain the PRRS, which can solve the problem of multicollinearity among the PRGs and simplify the risk score. In addition, we found that many of the PRGs included in the PRRS play an active role in LC progression and have their prognostic value through literature search, which has been stated earlier. Moreover, the PRRS can not only predict prognosis, but also guide therapeutic options in LUAD management.

## 5 Conclusion

In summary, this study constructed a PRRS which incorporated the expression of 14 PRGs and validated the predictive value of the risk score. Further investigation demonstrated the association between PRRS and TME, immunotherapy response and drug sensitivity, suggesting its potential for clinical application. Finally, a nomogram was constructed based on gender, age, tumor purity, TMB, PRRS, and stage, which achieved good accuracy in predicting prognosis.

## Supplementary Material

Supplementary Figure
