# Supplementary material for: Pyroptosis-based risk score predicts prognosis and drug sensitivity in lung adenocarcinoma
Source: Open Med (Wars). 2023 Mar 13;18(1):20230663. doi: 10.1515/med-2023-0663 (PMC10024350; doi:10.1515/med-2023-0663)
Supplement: Supplementary Figure [file med-2023-0663-sm.pdf]

Supplementary material

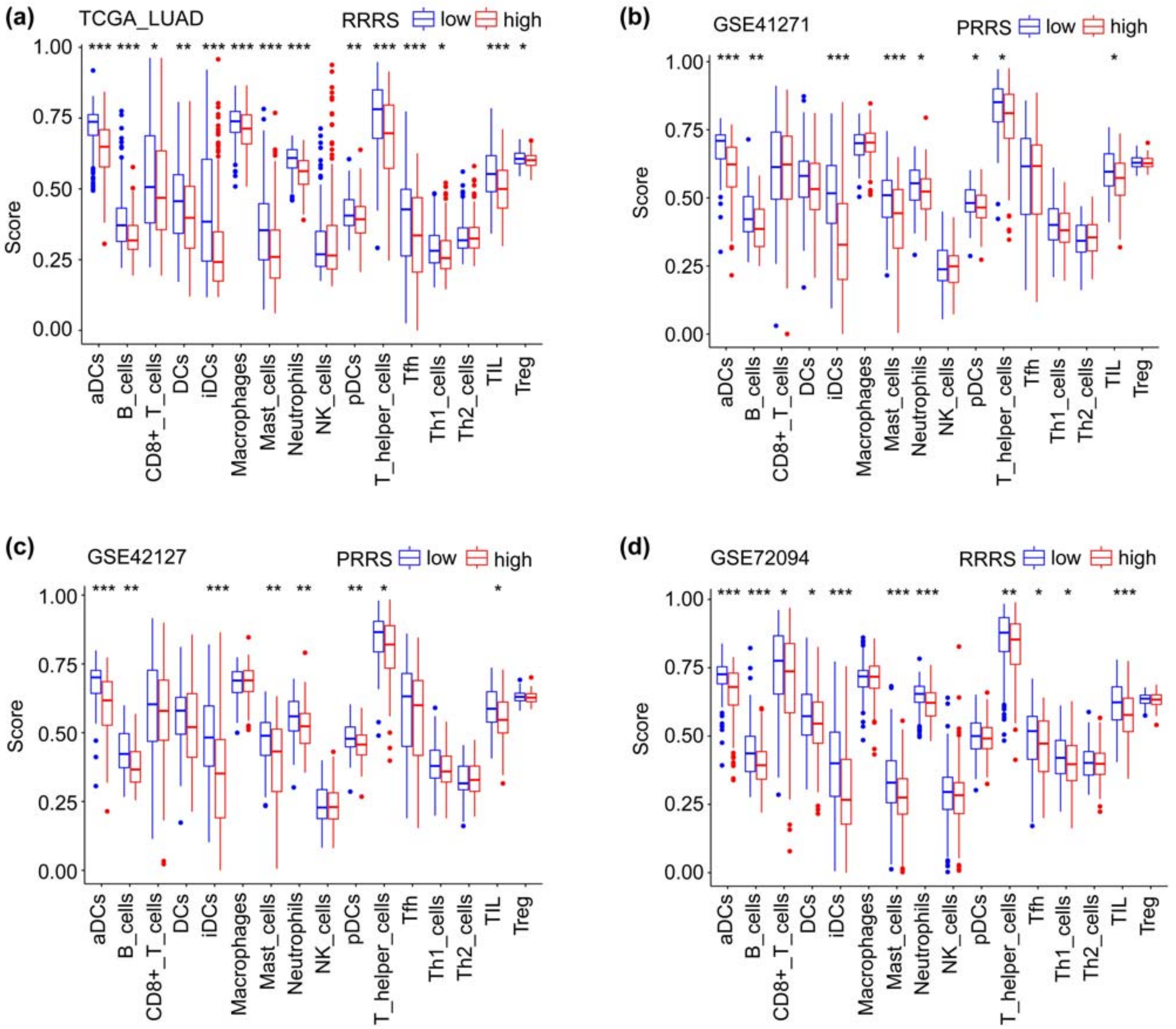

Figure S1: Boxplot of immune cell score based on PRRS classification. (a–d) Boxplot of immune cell score based on PRRS classification in TCGA_LUAD (a), GSE41271 (b), GSE42127 (c), and GSE72094 cohorts.

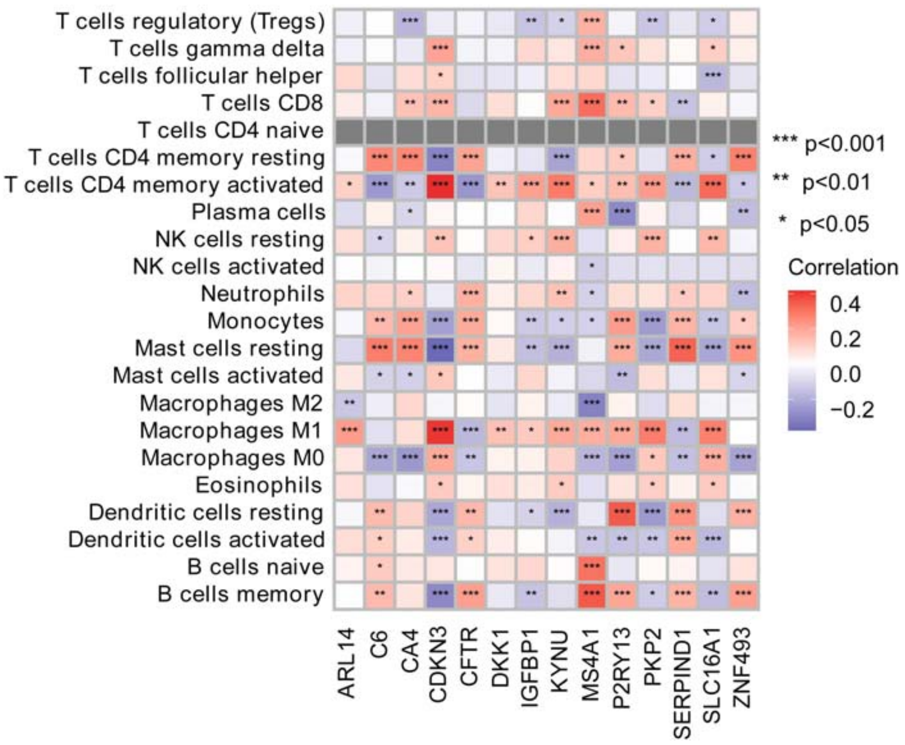

Figure S2: Heatmap of correlation between immune cells and the 14 crucial genes.

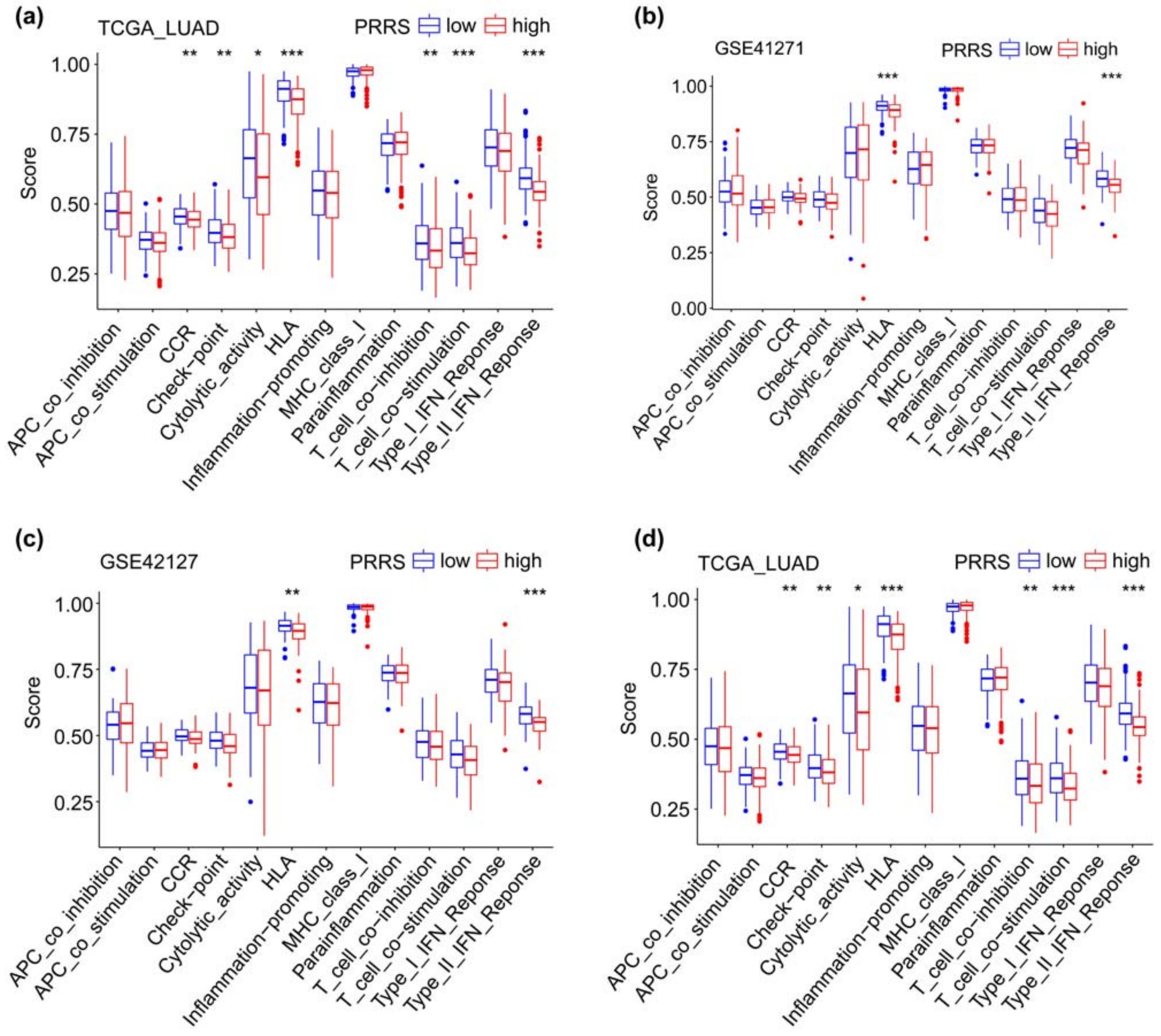

Figure S3: Boxplot of immune function score based on PRRS classification. (a–d) Boxplot of immune function score based on PRRS classification in TCGA_LUAD (a), GSE41271 (b), GSE42127 (c), and GSE72094 cohorts.

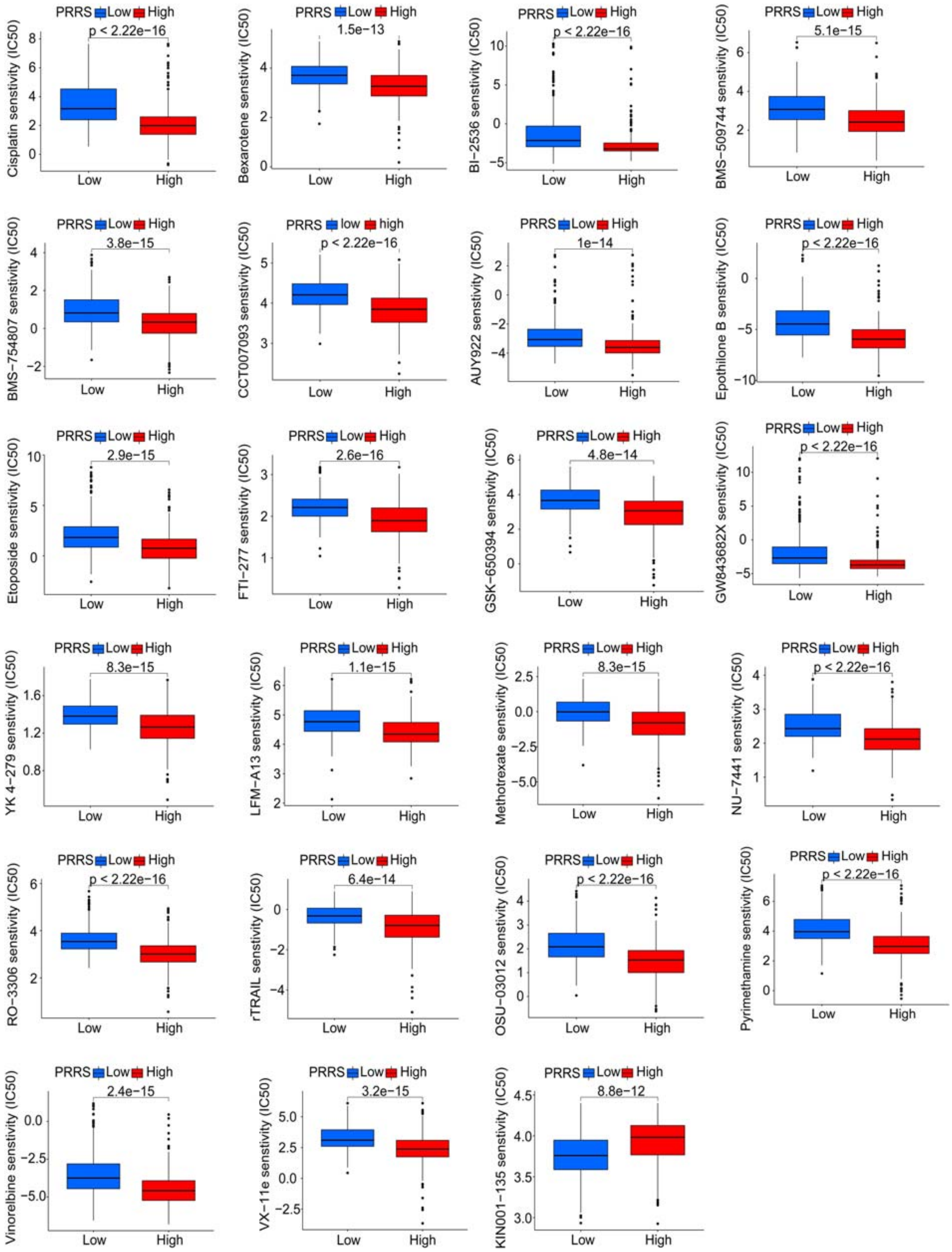

Figure S4: Boxplot of 23 drug sensitivity based on PRRS classification.

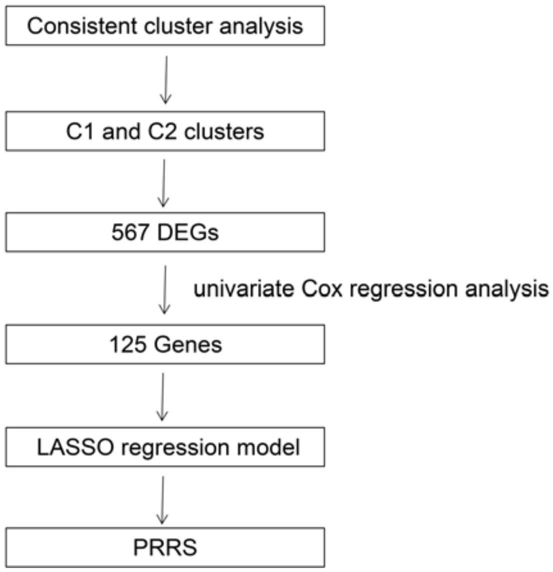
Consistent cluster analysis
C1 and C2 clusters
567 DEGs
univariate Cox regression analysis
125 Genes
LASSO regression model
PRRS

Table S1:

BAK1
BAX
CASP1
CASP3
CASP4
CASP5
CHMP2A
CHMP2B
CHMP3
CHMP4A
CHMP4B
CHMP4C
CHMP6
CHMP7
CYCS
ELANE
GSDMD
GSDME
GZMB
HMGB1
IL18
IL1A
IL1B
IRF1
IRF2
TP53

(*continued on next page*)

Table S1: continued

TP63
AIM2
CASP6
CASP8
CASP9
GPX4
GSDMA
GSDMB
GSDMC
IL6
NLRC4
NLRP1
NLRP2
NLRP3
NLRP6
NLRP7
NOD1
NOD2
PJVK
PLCG1
PRKACA
PYCARD
SCAF11
TIRAP
TNF
GZMA

Table S2:

Gene	ConMean	TreatMean	logFC	pValue
BAK1	8.6150235	16.04221472	0.896946691	3.22×10^{-18}
BAX	17.08344248	21.83901121	0.354308816	1.25×10^{-06}
CASP1	12.83620444	7.464538445	−0.782093711	2.26×10^{-16}
CASP3	8.076217981	14.52755871	0.847040529	9.54×10^{-24}
CASP4	10.20091341	12.62213294	0.307257385	0.004550071
CASP5	0.760562556	0.346921768	−1.132456548	3.57×10^{-18}
CHMP2A	42.61087278	49.83560199	0.225955154	0.001005167
CHMP3	19.26967426	14.5465664	−0.405653528	8.96×10^{-18}
CHMP4A	1.122536959	1.626156318	0.534703	1.85×10^{-07}
CHMP4B	85.58947852	129.9215684	0.602135591	8.59×10^{-19}
CHMP4C	6.89729913	15.60385562	1.177799113	7.95×10^{-20}
CHMP7	9.691000056	11.0582283	0.190402806	0.008576962
CYCS	17.50539741	29.8614096	0.770482455	1.35×10^{-12}
ELANE	0.475299506	0.507199286	0.093715813	1.32×10^{-10}
GSDMD	15.07083807	18.21831871	0.273630179	0.033781757
GSDME	1.172410131	1.851109339	0.658912773	0.028187444
GZMB	8.026075696	8.808986043	0.134281205	0.014819116
HMGB1	25.40562444	23.61271458	−0.105584017	0.000363331
IL18	11.0370835	9.026283697	−0.290154968	7.42×10^{-05}
IL1A	2.150229428	1.210965391	−0.828332969	1.19×10^{-18}
IL1B	5.503899663	3.151827233	−0.804265714	3.76×10^{-12}
IRF1	18.12491994	12.61495556	−0.522839499	3.29×10^{-07}
IRF2	15.4474917	14.50503334	−0.090818987	0.019966926
TP53	10.9817717	17.12177413	0.640721374	5.74×10^{-08}
AIM2	0.772349802	5.994535736	2.956321718	1.06×10^{-13}
CASP6	6.638925481	14.10219927	1.086898508	8.08×10^{-27}
CASP8	4.308892722	6.429552764	0.577401208	1.35×10^{-12}
GPX4	96.29079019	128.5634528	0.417010859	2.20×10^{-05}
GSDMA	0.326864169	0.700772503	1.100254932	5.01×10^{-05}
GSDMB	1.439805731	4.989170647	1.792925849	2.81×10^{-17}
GSDMC	0.450552611	3.054630501	2.761230392	1.63×10^{-15}
IL6	27.56644984	4.304621838	−2.678955074	5.88×10^{-13}
NLRC4	4.817283963	1.290919194	−1.899821272	9.44×10^{-30}
NLRP1	3.403277852	2.588114088	−0.395023726	2.36×10^{-06}
NLRP3	2.137975176	1.329290529	−0.685588648	2.61×10^{-07}
NLRP7	0.043459226	0.193659625	2.155788807	1.40×10^{-06}
NOD1	4.764992481	4.034663257	−0.24002567	1.65×10^{-05}
PJVK	0.385584956	0.639981053	0.730980432	7.20×10^{-05}
PLCG1	6.801641315	8.207382129	0.271039198	0.046550628
PRKACA	14.02464902	11.5622484	−0.278542694	6.38×10^{-09}
PYCARD	16.83378824	13.43138414	−0.325751889	0.000182721
TIRAP	2.045482704	2.391299498	0.225353493	0.03087598
TNF	1.721281976	1.451640005	−0.245799734	0.000511848
